# Tuning Mechanical and Self-Healing Properties Using Multivalent Crosslinking

**DOI:** 10.1021/acs.macromol.5c02522

**Published:** 2026-01-15

**Authors:** Sreecharan Ajjagola, Alexis K. Smith, Dominik Konkolewicz, Mehdi B. Zanjani

**Affiliations:** † Department of Chemistry and Biochemistry, 6403 Miami University, Oxford, Ohio 45056, United States; ‡ Department of Mechanical and Manufacturing Engineering, Miami University, Oxford, Ohio 45056, United States

## Abstract

Dynamic crosslinking in polymer networks has played a major role in contributing to various material properties, including toughness, tensile resistance, and self-healing. Dynamic covalent crosslinking, which connects two points on the polymer backbone using divalent crosslinkers, has been studied to date. Here, we systematically investigate the impact of using multivalent crosslinkers on the mechanical and self-healing properties of polymer materials. We used the thiol-Michael “click” chemistry to crosslink thiol–maleimide functional groups, which are well known for their thermoresponsive dynamic properties. Di-, tri-, tetra-, and hexathiols were used as crosslinkers to increase the complexity of the crosslinked polymer network. The results indicated that the mechanical and self-healing properties can be tuned by using multivalent networks, potentially paving the way for the development of better self-healing elastomers and opening opportunities for new chemistries to be explored.

## Graphical abstract

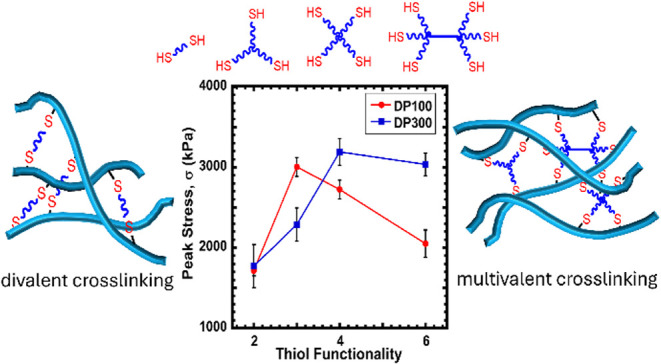

## 1 Introduction

Dynamic crosslinking is a powerful technique to achieve complexity in polymer architecture. It has significantly imparted various characteristic properties to polymeric materials and enhanced their mechanical performance.^1–6^ Over the last couple of decades, dynamic bonds made of noncovalent bonds like hydrogen bonds, electrostatics, van der Waals forces, hydrophobic effects, and pi and aromatic stacking, which form weak exchangeable interactions, have been incorporated into polymer networks.^7,8^ The dynamic crosslink networks formed by covalent bonds are called covalent adaptable networks (CANs), which contain functional groups that exhibit bond exchange and equilibrate. The reversible nature of the dynamic bonds introduces properties such as self-healing, reprocessability, recyclability, and degradability. In many cases, dynamic covalent bonds become active in response to stimuli such as changes in pH, temperature, and even light.^9,10^

The thiol-Michael reaction provides high yields and can be tuned using substituents, pH, solvent, and temperature.^11–16^ This base-catalyzed reaction is known for its excellent conversion with minimal byproducts. The thiol-Michael reaction has been proposed to follow a series of mechanistic steps, the first of which is the formation of thiolate by deprotonation, then the attack of the thiolate at the maleimide pi-bond, and the final step involves proton abstraction from a new thiol molecule by the enolate adduct.^17–19^ Due to its ease of implementation, mild reaction conditions, and reversibility at elevated temperature, thiol-Michael click chemistry has been extensively used as a dynamic crosslink.^9,20^ This study uses the dynamic bonding of thiol–maleimide chemistry as a model dynamic crosslink.

The exchange of rapid and fast-equilibrating noncovalent bonds and stimuli-responsive covalent bonds can help precisely control the macromolecular architecture of materials, enabling the exploration of a wide range of applications.^7^ Many of the crosslinkers studied to date have two ends or two sites that bond at two possible binding sites on the backbone polymer chains. The use of complex crosslinking agents may offer significant opportunities for augmenting the diverse characteristics of polymer materials.^9,11,19,21–23^ For instance, it has been demonstrated that adding carbon nanotubes or nanoparticles as crosslinkers improves the mechanical, electrical, and thermal characteristics of materials.^24–27^ Here, a complex bonding mechanism between the polymer backbones and carbon nanotubes introduces new properties into the composite materials. Multivalent crosslinking can greatly increase the performance of dynamic materials. This is because only two polymer chains are connected by a classical divalent crosslinker, whereas a crosslinker with multiple ends can bond to many backbone polymer chains, thereby potentially enhancing their structural and material properties.^27^ However, very high valencies of crosslinkers may be subject to challenges of steric hindrance and inefficiency of crosslinking.

A prior computational study was conducted by Hayes et al. that incorporates graph theory and molecular dynamics. They used the term “multi-arm crosslinkers,” indicating that the crosslinker has multiple sites radiating from a core that can be bonded to the polymer backbone via covalent bonds. The mechanical and self-healing properties of these polymers were investigated by considering two categories of multiarm crosslinkers using techniques that involved molecular dynamics simulations and graph theory. The study considered the angular stiffness and connectivity to assess the strength of the polymer network. The graph characteristics were compared with those of the properties of the material. Self-healing behavior and shape-dependent mechanical performance were evaluated based on different multiarm approaches to crosslinkers, indicating that intermediate valency of crosslinks led to superior mechanical properties.^6^ Inspired by this computational work, here we use crosslinkers with varying numbers of thiol groups as tunable multivalent thiol crosslinkers in the polymer networks. The material properties are studied as a function of the number of thiols per crosslinker in thiol–maleimide dynamic networks. crosslinker valency was controlled using commercially available multivalent thiols of divalent 2,2′-(ethylenedioxy)­dithanethiol, trivalent trimethylol tris­(3-mercaptopropionate), tetravalent pentaerythritol tetrakis­(3-mercaptopropionate), and hexavalent dipentaerythritol hexakis­(3-mercaptopropionate) (Scheme 1).

**Scheme 1:**
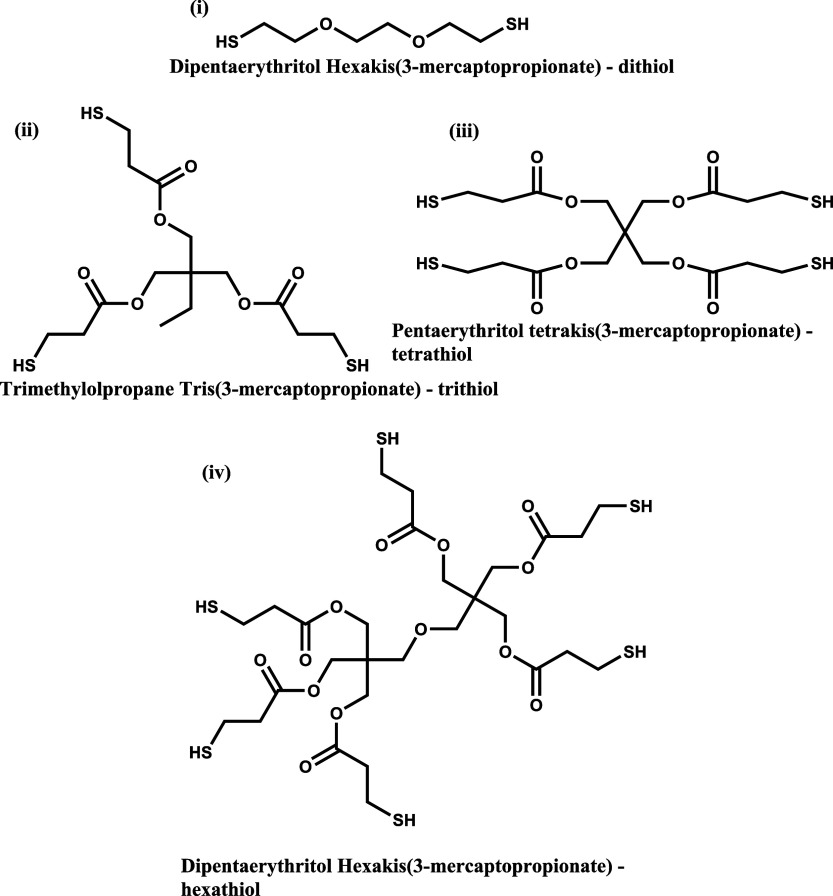
Multivalent Thiol Crosslinkers

## 2 Results and Discussion

In the computational study by Hayes et al., the multiarm crosslinks were evaluated, where an increasing number of crosslinking sites improved the mechanical behaviors of the materials. However, the complex multiarm crosslinkers can cause steric strain by bringing many polymers close together, which would, in turn, negate the effect of noncovalent bonds in the polymer architecture.^6^ We were able to construct similar polymer networks to those in the computational study by Hayes et al. Multivalent thiol crosslinkers used in our polymer networks have arm lengths comparable to those of the model constructed in the molecular dynamics simulation. The multivalent thiol crosslinkers we chose are flexible enough to find crosslinking on the polymer backbones.

**Scheme 2:**
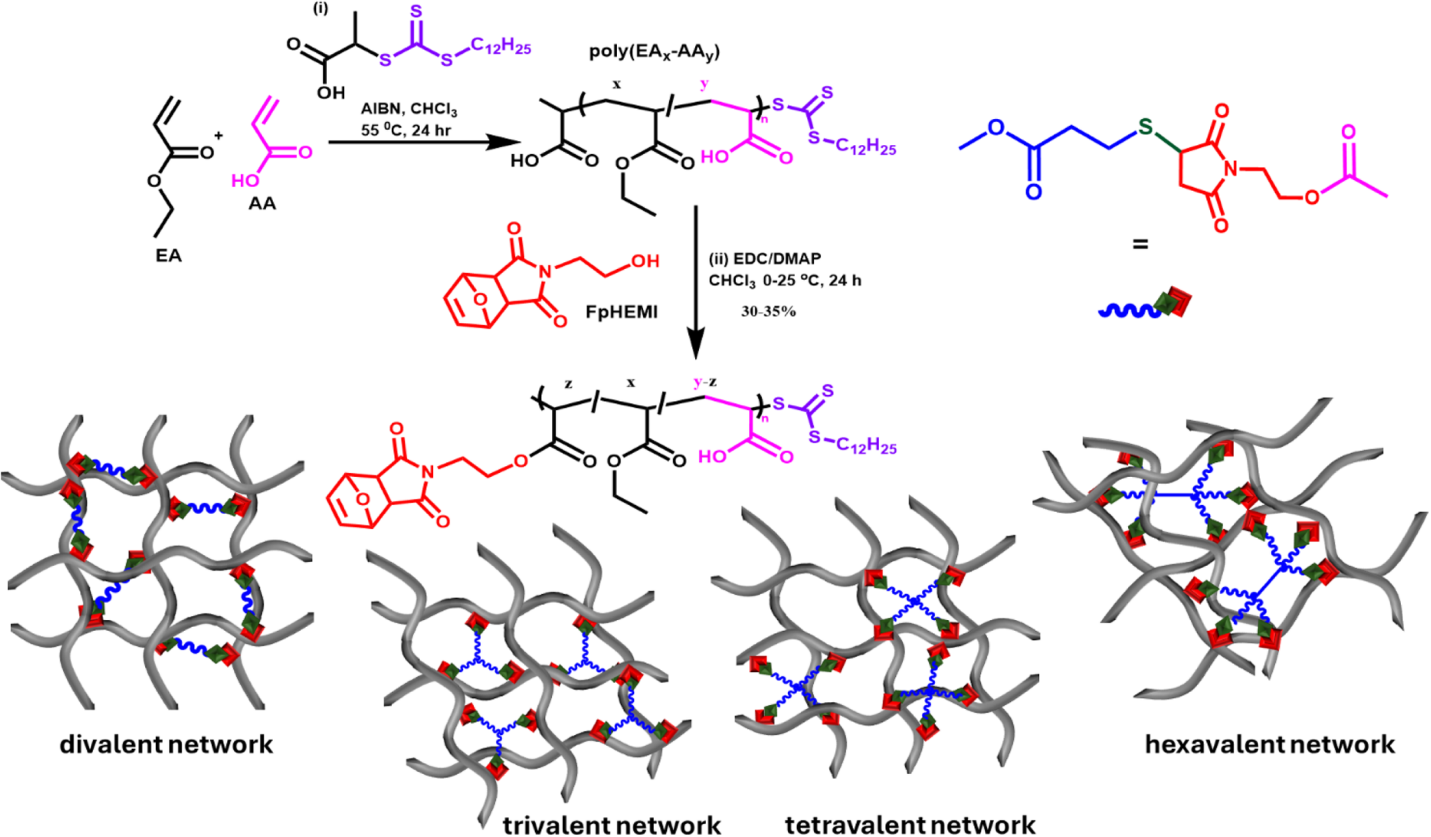
A General Method for Synthesizing Multivalent Polymer Networks with Thiol Crosslinkers (Blue) Bonded to Maleimide Groups on the Polymer (Red)

Thiol–maleimide (TM) crosslinks were incorporated into RAFT-polymerized ethyl acrylate and acrylic acid copolymers by functionalizing the carboxylic acid to an ester with a maleimide as a pendant group, as shown in Scheme 2. This furan-protected N-(2-hydroxyethyl) maleimide compound (FpHEMI) is synthesized using a known method from the literature.^28^ The coupling of the FpHEMI and acrylic acid on the polymer yielded 6% and 4% TM crosslinking densities, as the reaction functionalized 30% of the acid groups. Size exclusion chromatography (SEC) data are shown in Figure S4, which gives a representation of molecular weight averages of the polymer networks before and after the coupling with FpHEMI. Materials of two different average chain lengths, 100 (DP_100_6%) and 300 (DP_300_6%), were synthesized, and DP100 chains with two different crosslink densities of 4%(DP_100_4%) and 6% (DP_100_6%) were evaluated (Table S1). However, the divalent and the hexavalent linkers did not uniformly crosslink DP_100_4% polymers to form rigid enough elastomers to be subjected to DMA or the tensile tests. The lower number of maleimides, at 4% crosslinking, available to crosslink per chain decreases the probability of effective crosslinking, hence lowering the gel fraction and the network density. This results in a polymer network that has not achieved a gel point, or it ends up with a loosely crosslinked gel.^29^ As explained by the computational study, increasing the crosslinking sites will increase the probability of a strong network formation initially, which can be seen in the trivalent system, and further a decrease in strength can be seen with tetravalent systems, and then hexavalent systems fail to achieve complete gel formation as the probability of having more loose ends increases. Therefore, for DP_100_4%, only the trivalent and tetravalent linkers were studied, while, for DP_100_6% and DP_300_6%, divalent, trivalent, tetravalent, and hexavalent linkers gave rubbery materials that can be studied by a range of thermomechanical methods.

The thiol-based multivalent crosslinkers form the TM bonds, catalyzed by a base, leaving unreacted acrylic acid that can form hydrogen bonds. The dynamic TM crosslinking and the hydrogen bonding in materials have previously achieved dynamic properties in materials. The materials were heated at 110 °C for 10 h to induce crosslinking in a post-polymerization crosslinking approach.^30^ The amount of multiarmed thiol crosslinkers was added based on the number of pendant FpHEMI groups present on the polymer chains and thiol valency, ensuring the number of covalent crosslinks on the backbone polymer was constant for the materials made with thiol crosslinkers of different valencies. In addition to the TM bonding, we also expect the unfunctionalized acrylic acid groups to be involved in hydrogen bonding, which can be attributed to what can be termed as residual hydrogen bonds.^31,32^ The mechanical and thermal properties of the materials were evaluated by using dynamic mechanical analysis (DMA), tensile, and differential scanning calorimetry (DSC) experiments.^33^ Table S2 lists the properties of all the materials tested.

The DSC revealed that the *T*_g_s of the materials was slightly below room temperature, indicating the elastomeric nature and the rigidity of the materials due to an excess of acrylic acid^32,34^ groups on polymer backbones (Figure S3). Tensile tests of the materials were used to determine their toughness (Φ), peak stress (σ_peak_), and strain at break (ε_break_). To further understand the efficiency of the dynamic nature of TM bonds and the residual hydrogen bonds of acrylic acids as a function of crosslinker valency, self-healing experiments were performed with materials cut and heated at 110 °C for 10 h.

The frequency sweeps of all the materials were performed to understand the dynamic properties of the materials. For classical elastomers, the storage modulus (*E*′) of the materials tends to form a rubbery plateau at lower frequencies.^8^ According to the Affine network model, the modulus should be independent of crosslinker functionality/valency and should reduce with the phantom network model.^35^ In this case, the plateau modulus is given by^33^

1 $${E^{\prime }}_{\mathrm{plat}}=\frac{3\rho RT}{M_{c}}$$

where ρ is the polymer density, *R* is the universal gas constant, *T* is the absolute temperature, and *M*_c_ is the molar mass between crosslinks in the network. Within this framework, as long as the spacing of crosslinks along the backbone remains unchanged, the plateau modulus should be unchanged, regardless of crosslinker functionality.

However, in Figure 1 and Figure S2, the position of the plateau modulus and general *E*′ and *E*″ clearly depends on the crosslinker functionality. The general trends suggest that the modulus increases up to a certain crosslinker valency before reaching a maximum and then slowly decreasing or staying nearly constant at higher crosslinker valencies.

**Figure 1:**
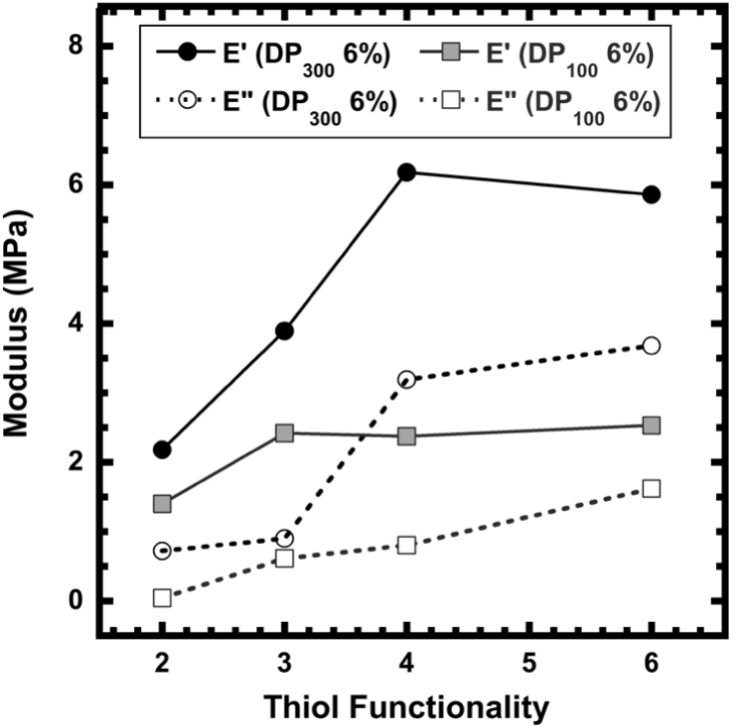
Storage (*E*′) and loss modulus­(*E*″) of multivalent polymer networks of chain lengths 100 (DP_100_6%) and 300 (DP_300_6%) at frequency 0.01 Hz.

Though only the divalent crosslinker plateaus at the lowest frequency achievable (0.01 Hz) by the DMA, the modulus at that frequency was considered for the other multivalent network materials as they approached a plateau.

In the DP_100_6% materials, polymer networks crosslinked by the trithiol linker had the highest value of storage modulus (*E*′), with no significant increase in their values with an increase in the valency of the crosslinkers. However, in the DP_300_6% materials, the modulus value peaked for materials with tetrathiols in their polymer network and then decreased somewhat with the hexathiol linker. The materials with increased chain lengths could effectively have more elastic connections and entanglements per chain than in the lower chain length networks, which shifts the highest *E*′ value to the tetrathiol networks. The storage (*E*′) and loss­(*E*″) moduli are compared to understand the viscoelastic nature of the materials. The storage modulus *E*′ indicates the elastic nature, which tends to increase up to the peak with the tri/tetravalent crosslinker, followed by a near plateau. This is because the multivalent crosslinker leads to more elastically effective crosslinks, with fewer ineffective loops or dangling chains, which leads to an increase in storage modulus.^8,33^ The entanglements due to an increase in chain lengths also increased the *E*′ values when the chain length was increased from 100 to 300. However, within the same network density and chain length, the increase in valency affects increasing modulus values to an extent. Then, with a further increase in valency of the thiols, for example, in polymer networks with hexathiols, various factors, such as entanglements, un-crosslinked loose ends, and oxidized thiols, can interrupt effective crosslinking. Therefore, we do not observe any increase in the value of *E*′, although we see their viscous response, i.e., *E*″, increase with an increase in crosslinking valency, which makes energy dissipation more effective. This is because of the complex nature of the crosslinkers. The more constraints created by the increasing valency force the chains to slide with higher friction, leading to an increase in *E*″, unlike *E*′. As evidence that higher entanglements can undergo segmental rearrangements that dissipate energy, we see an increase in *E*″ with chain length. But clearly, our systems indicate that at room temperatures *E*′ > *E*″ which suggests a solid-like material. This is especially true at lower frequencies, where there is a higher damping effect in multivalent networks.^33^

Following the DMA analysis, which focuses only on small amplitude deformations, we expanded to tensile tests and compared the peak stress (σ_peak_), strain at break (ε_break_), and toughness (Φ) of the materials as they changed with different thiol functionalities in DP_100_6% and DP_300_6% materials.

The σ_peak_, ε_break_, and Φ of the materials revealed a trend similar to the storage modulus values, as observed in the computational model of the multivalent network. In the DP_100_6% materials, trithiol materials had the highest values of σ_peak_, ε_break_, and Φ, and each parameter decreased for tetrathiol and hexathiol. Despite having a similar number of linkages between the crosslinkers and the polymer backbones, a trend is evident here as the valency of the thiol crosslinkers increases. This could be associated with the polymer network structure affected by the noncovalent bonding between the polymer chains. Prior molecular dynamics simulations also show that the polymer chains tend to be intertwined with a six-arm crosslinker in the network, thereby negating the effect of any noncovalent bonding as the polymer chains come into close proximity.^6^

The material toughness (ϕ) given by the area under the stress–strain curve also showed that trithiol materials are tougher, having more noncovalent bonds than tetrathiol or hexathiol materials. The peak stress and the strain at break of the DP_100_6% materials also evidenced the change in proximity of the polymer chains for tetrathiol and hexathiol materials, as the storage modulus did not have much effect, despite the materials showing somewhat lower resistance to applied stress. This can also be explained by the angular stiffness factor, which is the ratio of the angle between bonds in the crosslinker arms to the angle within polymer chains, as described in the graph model constructed.^6^ Although the strength of the materials increases initially with an increase in stiffness, which is directly dependent on the angles in the crosslinkers, excessive stiffness results in a drop in mechanical strength due to the inability of the crosslinkers to form bonds, which is attributed to a steric effect. The extensibility of the materials provides further proof for the crosslinkers affected by stiffness, as the strain at break (ε_break_) also increases for the trithiol crosslinkers when compared to the dithiol, but tends to decrease with higher crosslinker valency.

This general trend persisted but shifted slightly when the chain length of the materials was increased. The tetrathiol materials showed the highest strength, followed by a decrease with the hexathiol materials (Figure 3). This can again be the case with polymers that have a longer chain length, which will have more flexible connections and more acid groups in the chain, increasing the residual hydrogen bonding. It is important to note that materials with hexathiol crosslinkers invariably either decrease or show no improvement in their mechanical properties.

The dynamic exchange of the thiol–maleimide bonds enables self-healing in the polymer networks.^8^ To activate the dynamic exchange, materials are healed at 110 °C. Figures 2a and 3a present the stress–strain curves for the self-healed materials. Based on the computational model, it was expected that self-healing would improve with an increase in the valency of the crosslinkers, with the hexathiol crosslinked materials having the highest recovery and the dithiol being the least. This is because having a higher number of crosslinking sites would mean higher chances of finding and restoring a crosslinking site during bond exchange. However, in the DP_100_6% materials, the dithiol crosslinked networks showed a recovery in strain of 94% and 76% of the stress, while the trivalent and tetravalent networks showed recoveries of 31% and 63%, and 45% and 78%, respectively, in terms of strain and stress. The DP_300_6% materials showed a similar trend, although the recovery was lower compared to the DP_100_6% materials. The hexathiol crosslinked networks, which also have lower toughness values, as depicted in Figure 4, exhibit a higher recovery percentage, similar to the dithiol crosslinked materials. This is, in fact, consistent with the previous study on crosslinkers, as the rigidity of the polymer network increases due to higher crosslink densities, the exchange of dynamic bonds can be inhibited.^8,9^ The trivalent networks of DP_100_4% materials with recovery in strain of 99% and 90% of the stress (Table S4) further support this, as both trivalent and tetravalent networks of reduced crosslink densities showed a great stress–strain curve as observed in Figure S1.

**Figure 2:**
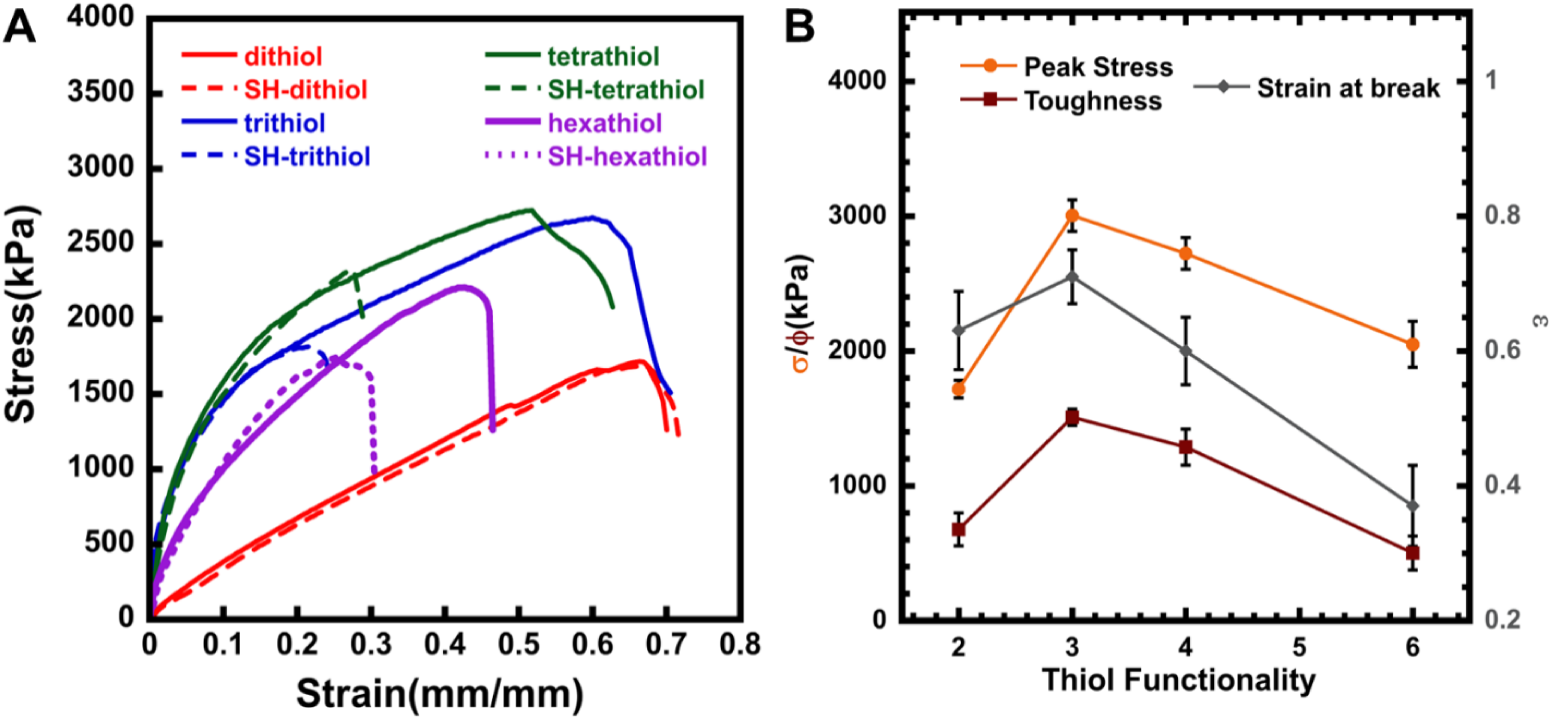
(A) Stress–strain curves for pristine (solid lines) and self-healed (dashed lines) DP_100_6% multivalent crosslinked materials and their (B) peak stress, strain at break, and toughness compared with different thiol functionalities.

**Figure 3:**
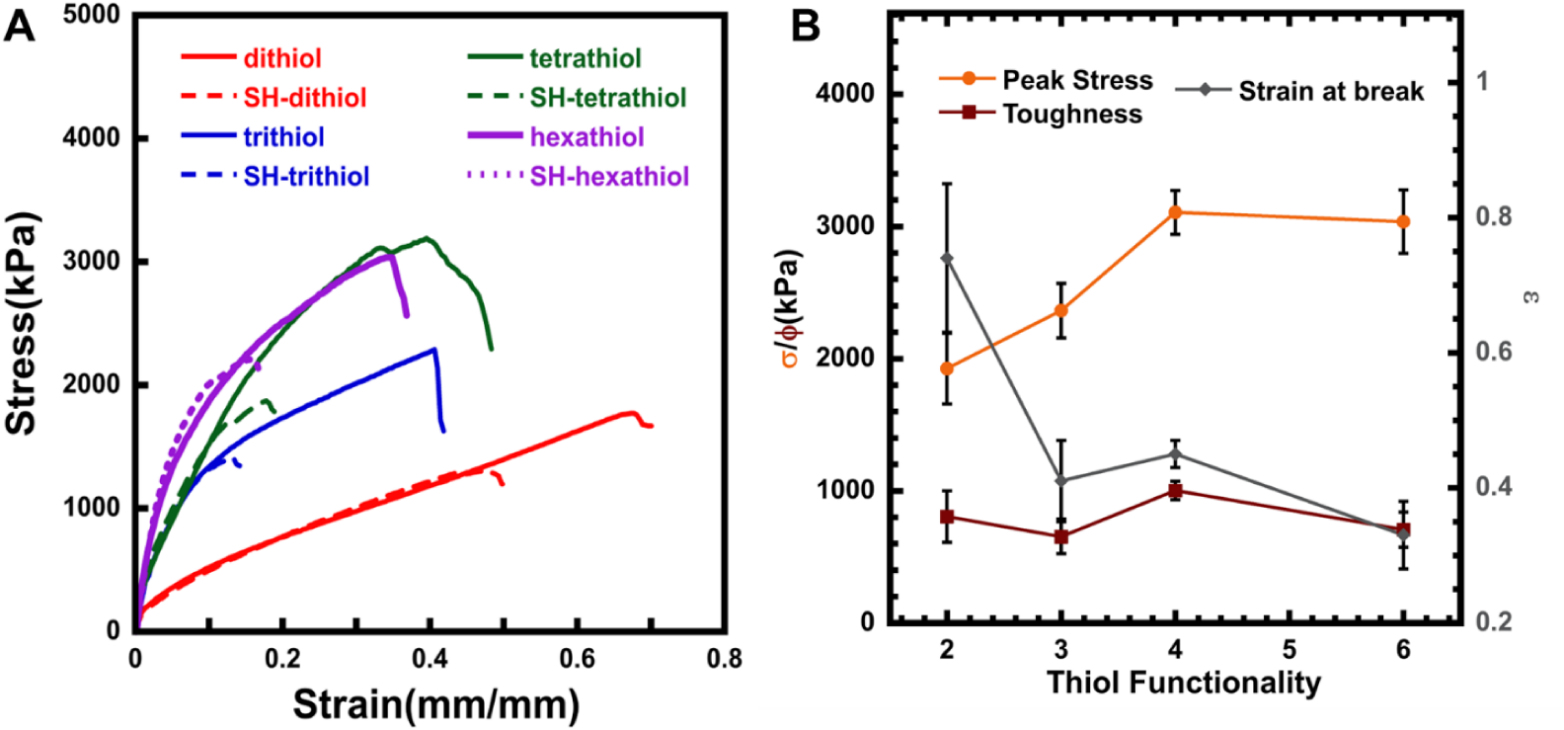
(A) Stress–strain curves for pristine (solid lines) and self-healed (dashed lines) DP_300_6% multivalent crosslinked materials and their (B) peak stress, strain at break, and toughness compared with different thiol functionalities.

**Figure 4:**
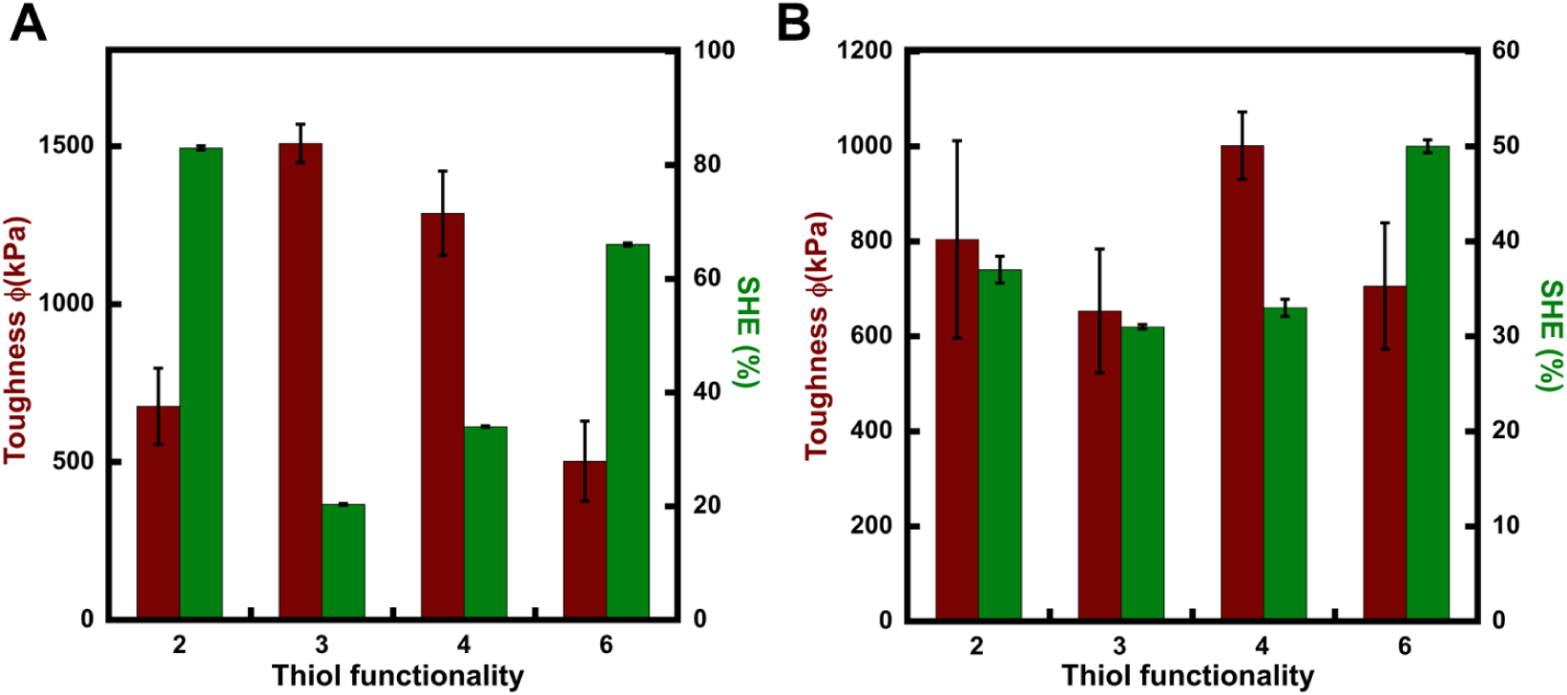
Self-healing efficiency with respect to toughness of the (A) DP_100_6% and (B) DP_300_6% materials.

## 3 Conclusions

In summary, multivalent thiol crosslinkers (di-, tri-, tetra-, and hexa-) were crosslinked with well-defined polymers containing pendant maleimides through thiol-Michael “click” chemistry. The model polymer networks constructed in this study showed that the material properties can be tuned by using crosslinkers of different valencies. The results established a clear dependence on the network architecture and valency of the crosslinkers. The valency of the crosslinker had an impact on its mechanical and self-healing properties, even with constant total crosslink density and polymer architecture. The toughness and the self-healing efficiency graphs support the tunability of the self-healing properties, as they confirm that in the studied systems the self-healing ability deteriorates with an increase in toughness. Moreover, the system studied here also factors in the noncovalent bonding in the network that influences the combined mechanics of the system. Overall, the multivalent crosslinked polymers create a complex network tunable by using crosslinkers of different functionalities and valencies. Although it is worth mentioning that within the framework presented here, parameters such as peak stress, toughness, and self-healing could be easily tuned, other parameters, like the noncovalent bonds and the exact values of angular stiffness, are harder to directly control in experiments. The study presented here can also be applied to future studies on creating accurate models of the crosslinking agents according to the experimental guidelines established here and exploring the large parameter space that is available to such multivalent network structures through data-driven approaches. Our work has established practical parameters that may be varied and tuned to enhance the structural, mechanical, or self-healing properties of such crosslinked polymer materials. Further, different chemistries can be utilized for multivalent networks, as we have clearly established that the materials can be better tuned by increasing the degrees of freedom in crosslinkers.

## 4 Experimental Section/Methods

### 4.1 Preparation of Poly­(EA_*x*_AA_*y*_+FpHEMI)

The process of making the materials began with the synthesis of Poly­(EA_*x*_AA_*y*_) (ratios, EA:AA −80:20, 240:60, and 85:15) polymer using RAFT polymerization. As shown in Scheme 1, polymerization was conducted with chloroform as the solvent. PADTC, synthesized as described in the literature,^36,37^ was selected as the RAFT agent, and AIBN as the initiator. Ethyl acrylate (EA) was used as a monomer for the backbone to obtain a good flexible polymer with the glass transition temperature being below the room temperature, which helps support the dynamic properties.^24,25^ The next step in forming our polymer backbone was the addition of a furan-protected maleimide group. For this, furan-protected N-(2-hydroxyethyl) maleimide (FpHEMI) was used and synthesized as given in Scheme S1.

### 4.2 Crosslinking

The amount of the thiol-crosslinkers used was calculated based on the percentage of yield obtained in the coupling process. The crosslinkers were added to the precipitated coupled polymer after dissolving it in DMF solvent. The mixtures were poured into Teflon molds, tributylamine base was added, and then they were placed on hot plates for about 24 h. The crosslinked polymer networks begin to turn red, indicating the formation of thiol–maleimide crosslinks. The materials are placed inside an oven at 110 °C for 10 h before being subjected to mechanical analysis.

The typical deprotection of the furan group after the coupling reaction and before crosslinking would require additional steps, involving the reflux of the coupled reaction for 48–72 h to eliminate the protected furan, followed by precipitation. A previous study from our laboratory on the use of multiple types of dynamic bonds reported the formation of a thiol-Michael adduct due to the addition between thiol groups and 1,1′-(methylene di-4,1-phenylene)-bismaleimide (BMI). BMI was used here as a crosslinker to form a Diels–Alder (DA) bond with the furan group, thereby forming a thiol–maleimide moiety at the free thiol group generated by the ethyl xanthate ethyl acrylate monomer. To evaluate the two reactions, a small molecule study was conducted by using *N*-methyl maleimide and furfuryl alcohol, which were subjected to form a DA adduct in the presence of 2-mercaptoethan-1-ol at 90 °C. The ^1^H NMR spectrum of this mixture confirmed the formation of furfuryl alcohol and the thiol-Michael adduct, which proves that the equilibrium strongly favors the formation of the thiol–maleimide bond over the DA product. This transition occurs through the retro-DA reaction of the DA adduct, which is followed by the thiol-Michael addition.^24^ With similar assumptions regarding an in situ reaction where the thiol-Michael adduct is formed preferentially over the DA adduct, we obtained materials that gradually crosslinked upon deprotection or evaporation of furan through a retro-DA reaction (Scheme S2). We predicted that it would be easier with this system, as the retro-DA product here gives furan, which has a lower boiling point of 31 °C.

### 4.3 Self-Healing

The materials, after 24 h of initial crosslinking, were cut in half with a razor blade, and the two separate parts were reassembled and pressed together, along with excess amounts of base added at the point of healing. At this point, both self-healed and pristine samples were placed in the oven at 110 °C for 10 h, as we exposed all the samples to equivalent thermal conditions before subjecting them to tensile tests.

### 4.4 Analytical Methods

Nuclear magnetic resonance (NMR) spectroscopy was conducted on a Bruker 400 MHz spectrometer. Size exclusion chromatography (SEC) was performed on an Agilent 1260 SEC system using dimethylformamide (DMF) as the eluent running at 1 mL per minute. Differential Scanning Calorimetry (DSC) was performed on TA Instruments DSC Q2000 using a heat–cool–heat cycle, with a temperature range of −40 to 70 °C. The heating rate was 10 °C per minute, and the data were collected from the second heating cycle. The frequency sweeps of the materials were conducted on a TA Instruments Q800 dynamic mechanical analyzer (DMA) in film tension mode to determine the storage and loss moduli of the materials.

### 4.5 Tensile Tests

An Instron 3344 universal testing system, equipped with a 100 N load cell, was used to perform tensile testing on both pristine and self-healed materials at room temperature. The materials were elongated at a rate of 0.05 mm s^–1^. The tests were conducted until the samples failed.

## Supplementary Material



## Notes

Supporting Information and methods, supplemental polymer molecular characterization data, polymer network mechanical data, polymer network thermal properties, and stress relaxation data (PDF)
