# Supplementary material for: Tuning Mechanical and Self-Healing Properties Using Multivalent Crosslinking
Source: Macromolecules. 2026 Jan 15;59(2):663–70. doi: 10.1021/acs.macromol.5c02522 (PMC12854739; doi:10.1021/acs.macromol.5c02522)
Supplement: Supplementary file 1 [file ma5c02522_si_001.pdf]

# Tuning mechanical and self-healing properties using multivalent crosslinking

Sreecharan Ajjagola,[a] Alexis Smith,[a] Dominik Konkolewicz,[a] Mehdi B. Zanjani [b]

[a]Department of Chemistry and Biochemistry and [b]Department of Mechanical and Manufacturing Engineering, Miami University, Oxford, Ohio 45056, USA

## Methods

### *Characterizations*

*Nuclear Magnetic Resonance (NMR) Spectroscopy*

$^1$H NMR spectra were obtained on a Bruker 400 MHz spectrometer. Monomer conversions were determined via proton ($^1$H) NMR spectroscopy. Data are reported in ppm using $CDCl_3$ as the solvent, unless otherwise specified.

*Size exclusion Chromatography (SEC)*

Molecular weights and dispersities were determined using an Agilent 1260 SEC system equipped with an autosampler, an Agilent 1260 isocratic pump, an Agilent 1 guard and 2 analytical Polar Gel-M columns, a degasser, an Agilent 1260 refractive index [RI detector, and a viscometer for universal calibration. N,N-dimethylformamide (DMF) + 0.1 wt.% LiBr was used as the eluent with a flow rate of 1 mL/min at 25 °C. The system was calibrated with poly[methyl methacrylate] standards with molecular weights in the range of 617500 to 1010. All samples were filtered through a 200 nm PTFE filter prior to injection.

*Differential scanning calorimetry (DSC)*

All glass transition temperatures [Tg] were obtained using the TA Instrument DSC Q2000. The data was obtained in a heat cycle ranging from -40 °C to 70 °C with a heating rate of 10 °C/min. Data from the second heating cycle was used to plot the curve.

*Dynamic Mechanical Analysis (DMA)*

All frequency sweep experiments were performed using the isothermal frequency sweep test method on the TA instrument DMA Q800 equipped with a tension clamp. Frequency ranged from 0.01- 100 Hz at a constant temperature of 30 °C. A strain of 0.1% and a preload force of 0.01 N was applied.

*Tensile testing on Instron*

An Instron 3344 universal testing system equipped with a 100 N load cell was used to conduct tensile testing of the materials at room temperature to obtain a stress-strain curve. The extension was increased at a rate of 0.5 mm/s. For all the samples, data was collected till the sample failed. Every tensile experiment was repeated twice.

## Materials

All chemicals used in the synthesis were purchased from commercial sources and used as received.

***Synthesis***

*Synthesis of 3a,4,7,7a-tetrahydro-4,7-epoxyisobenzofuran-1,3-dione (Furan-protected maleic anhydride (Fp-MAn))*

Maleic anhydride (MAn, 68.6g, 0.700 mol) was added to a round bottom flask containing a magnetic stirrer bar and dissolved in 350 mL of toluene. The reaction mixture was heated to 80 °C, where furan (76.3 mL, 71.4g, 1.05 mol) was added. The reaction mixture was capped with a glass stopper and cooled to room temperature. The reaction proceeded at room temperature for 24 hours, yielding a white precipitate. The precipitate was filtered and washed with diethyl ether. The white solid was collected to give Fp-MAn (90 g, 0.55 mol, 78% yield). The compound was confirmed by 1H-NMR in agreement with the literature.[1] $^1$H-NMR(400 MHz, $CDCl_3$) $\delta$ ppm 6.58 (2H, sing), 5.46 (2H, sing), 3.17 (2H, sing).

*Synthesis 2-(2-hydroxyethyl)-3a,4,7,7a-tetrahydro-1H-4,7-epoxyisoindole-1,3(2H)-dione (Furan-protected N-(2-hydroxyethyl) maleimide (FpHEMI))*

Fp-MAn (45.0 g, 0.271 mol) was placed in a round bottom flask containing a magnetic stirrer bar and dissolved in 150 mL of anhydrous methanol. The flask was capped with a rubber septum, deoxygenated by bubbling with nitrogen for 10 min, and then placed in an ice bath. To this solution, 2-aminoethanol (17 mL, 17.2 g, 0.281 mol) was added via a syringe. The reaction mixture was stirred at 0 °C for 30 min and then refluxed for 14 h. After reacting, the solution was cooled to room temperature and then cooled to -20 °C. The product Fp-HEMI crystallized out of solution at -20 °C. The solid was collected by filtration and washed with isopropanol and allowed to dry. The solid was collected to give Fp-HEMI(30.84, 0.15 mol, 55% yield). The compound was confirmed by $^{1}$H-NMR in agreement with the literature.[1] $^{1}$H-NMR(400 MHz, $CDCl_3$) $\delta$ ppm 6.56 (2H, sing), 5.31 (2H, sing), 3.76 (4H, multi), 2.92 (2H, sing), 2.08 (1H, sing).

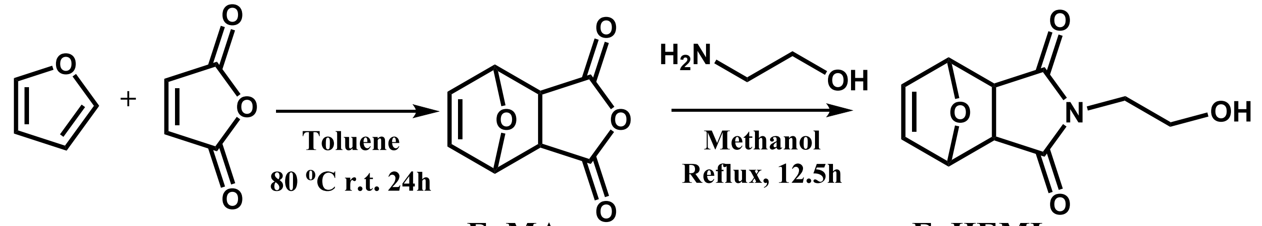


FpMAn FpHEMI

**Scheme S1. Synthesis of FpHEMI**

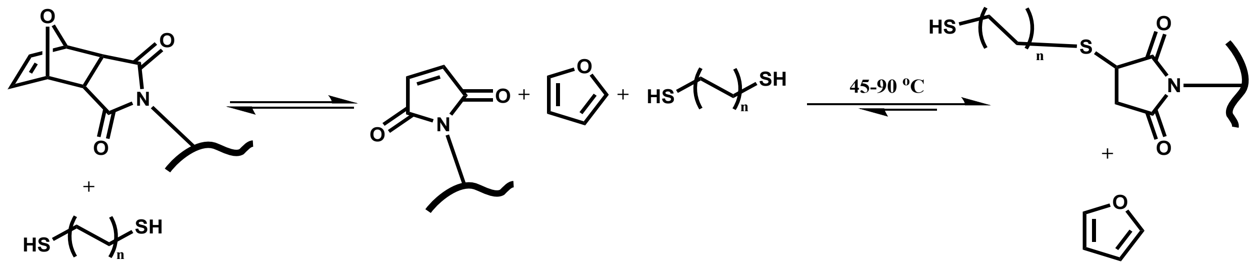


**Scheme S2. Proposed equilibrium for in situ deprotection and crosslinking**[2]

**Supplementary data:**

**Table S1.** Conversion in RAFT polymerization and coupling reactions, $M_n$ and Đ (from SEC) for polymers in this study.

| Polymers | Conversion (%) | $M_n$ | Đ |
|---|---|---|---|
| Poly($EA_{80}AA_{20}$) | >98 | 15402 | 1.1 |
| Poly($EA_{240}AA_{60}$) | 97 | 32422 | 1.2 |
| Poly($EA_{85}AA_{15}$) | 97 | 15155 | 1.1 |
| Poly($EA_{80}AA_{20-x}$) + $FpHEMI_x$ | 30 | 15752 | 1.2 |
| Poly($EA_{240}AA_{60-y}$) + $FpHEMI_y$ | 33 | 36105 | 1.3 |
| Poly($EA_{85}AA_{15-z}$) + $FpHEMI_z$ | 28 | 14967 | 1.2 |

**Table S2. Summary of the thermal and mechanical properties of the $DP_{100}$6% materials.**

| $DP_{100}$6% | dithiol | trithiol | tetrathiol | hexathiol |
|---|---|---|---|---|
| $T_g$ (°C) | 12.91 | 17.95 | 18.2 | 17.59 |
| E' (MPa) | 1.4 | 2.42 | 2.37 | 2.53 |
| E" (MPa) | 0.04 | 0.61 | 0.8 | 1.61 |
| σ (kPa) | 1739.5±66 | 2918.3±117 | 2770.5±118 | 1965.2±170.6 |
| ε (mm/mm) | 0.63±0.058 | 0.71±0.04 | 0.6±0.05 | 0.37±0.06 |
| SH(σ ) % | 0.76 | 0.63 | 0.78 | 0.9 |
| SH(ε ) % | 0.94 | 0.31 | 0.45 | 0.7 |
| Toughness (kPa) | 676.1±121.2 | 1508.3±60.8 | 1286.5±133.4 | 502.1±126.7 |

**Table S3. Summary of the thermal and mechanical properties of the $DP_{300}$6% materials.**

| $DP_{300}$6% | dithiol | trithiol | tetrathiol | hexathiol |
|---|---|---|---|---|
| Tg (°C) | 17.24 | 18.02 | 17.74 | 17.23 |
| E' (MPa) | 2.18 | 3.89 | 6.18 | 5.86 |
| E"  (MPa) | 0.72 | 0.9 | 3.19 | 3.68 |
| σ (kPa) | 1924.5±267.6 | 2362.3±207.2 | 3105.1±166.1 | 3035.5±239.9 |
| ε (mm/mm) | 0.74±0.11 | 0.41±0.06 | 0.45±0.02 | 0.33±0.05 |
| SH(σ ) % | 0.6 | 0.62 | 0.62 | 0.73 |
| SH(ε ) % | 0.57 | 0.6 | 0.62 | 0.6 |
| Toughness (kPa) | 803.9±208 | 653.3±130.65 | 1001.4±70.1 | 705.4±133.1 |

**Table S4. Summary of the thermal and mechanical properties of the $DP_{100}$4% materials.**

| $DP_{100}$4% | trithiol | tetrathiol |
|---|---|---|
| Tg (°C) | 17.5 | 17.07 |
| E' (MPa) | 2.28 | 1.24 |
| E"  (MPa) | 0.15 | 0.16 |
| σ (kPa) | 1033.4±27.5 | 935.6±26 |
| ε (mm/mm) | 0.7±0.01 | 0.47±0.07 |
| SH(σ ) % | 0.9 | 0.78 |
| SH(ε ) % | 0.99 | 0.84 |
| Toughness (kPa) | 440.1±15.1 | 267.2±40.7 |

**Table S5. Characteristic relaxation times, stretching exponents and average relaxation times for $DP_{100}$6% multivalent networks.**

| Networks | Dithiol | | Trithiol | | Tetrathiol | | Hexathiol | |
|---|---|---|---|---|---|---|---|---|
| Temperature | 150 °C | 170 °C | 150 °C | 170 °C | 150 °C | 170 °C | 150 °C | 170 °C |
| $\tau^*$(s) | 80000 | 2395 | 35000 | 3095 | 49888 | 3333 | 22143 | 726 |
| $\beta$ | 0.43 | 0.46 | 0.28 | 0.47 | 0.25 | 0.78 | 0.42 | 0.67 |
| $\langle\tau\rangle$ (s) | 216659 | 5733 | 381817 | 6988 | 1164438 | 3826 | 65052 | 961 |

$$\frac{G(t)}{G_0} = e^{[-\left(t/\tau^*\right)^{\beta}]} \quad \text{(S1)}$$

$$\langle\tau\rangle = \frac{\tau^*\,\Gamma(1/\beta)}{\beta} \quad \text{(S2)}$$

The stress relaxation profiles of multivalent networks are fitted to the Kohlrausch−Williams−Watts (KWW) stretched exponential decay function where, $G(t)/G_0$

is the normalized shear modulus at time t, $\tau^*$ is characteristic relaxation time, $\beta$ is stretching exponent ($0 \leq \beta \leq 1$).[3] The average relaxation time, $\langle\tau\rangle$, is obtained from the fitted KWW parameters of $\beta$ and the characteristic relaxation time, $\tau^*$ in which $\Gamma$ is the Gamma function.[3,4]

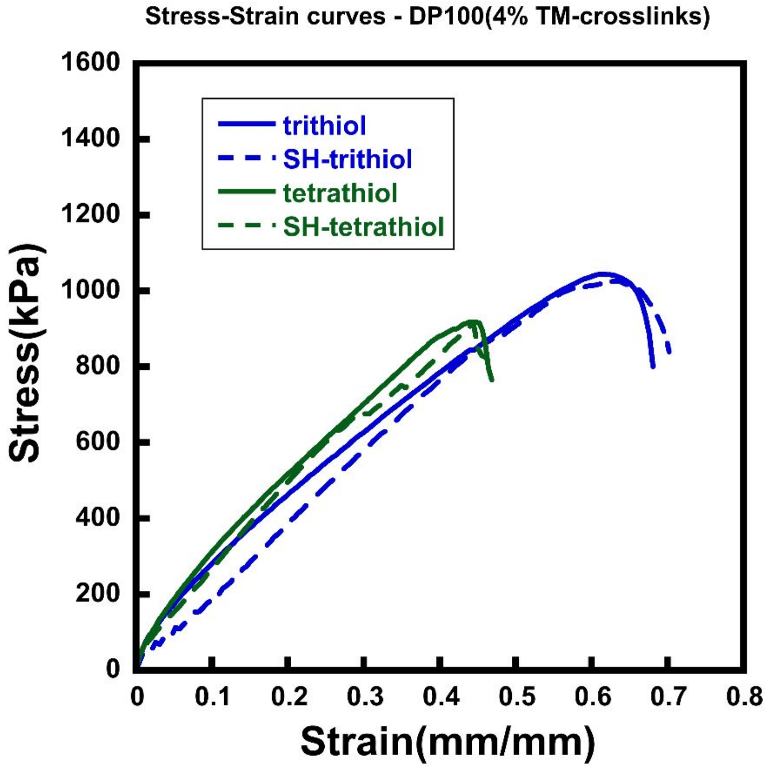


**Figure S1. Stress-strain curves for pristine and self-healed $DP_{100}$4% multivalent crosslinked materials.**

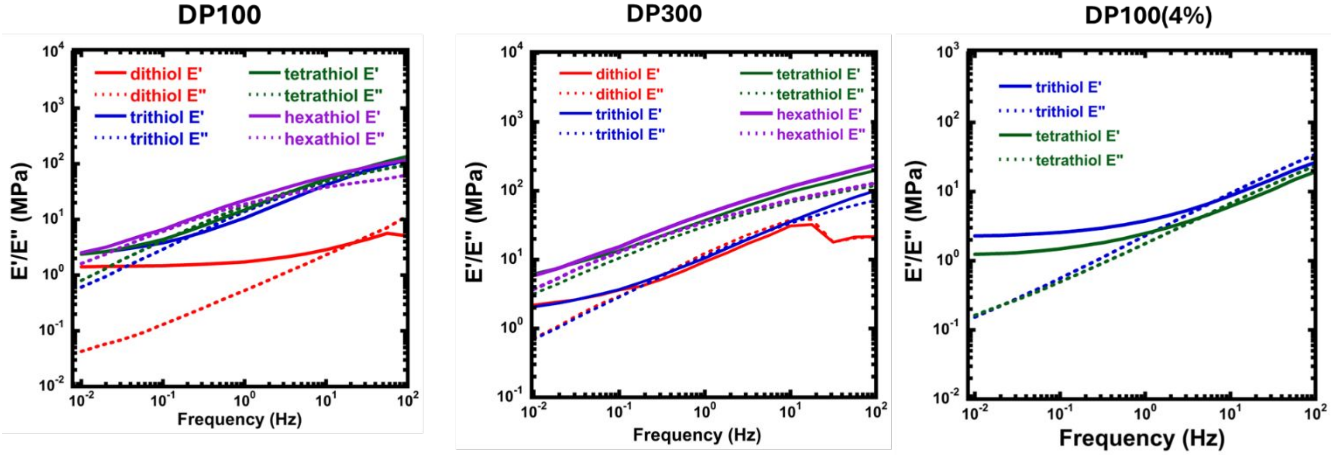


**Figure S2. Frequency sweep results for the $DP_{100}$6%, $DP_{300}$6%, $DP_{100}$4% materials, where the DP label refers to the degree of polymerization. Storage(E') and Loss modulus(E") are shown for the experiment conducted at 30 °C.**

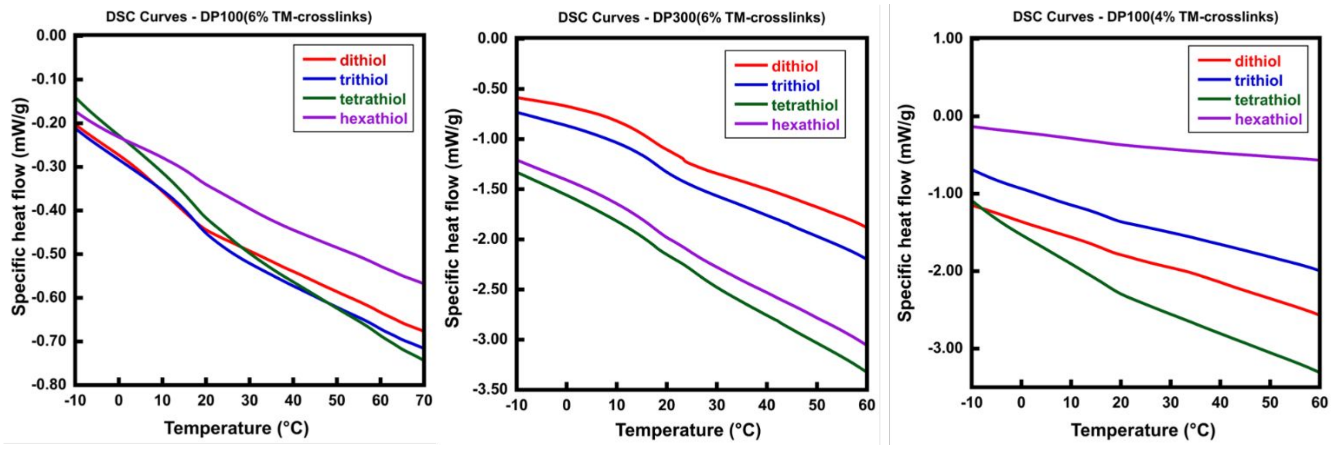


**Figure S3. DSC curves for the $DP_{100}$6%, $DP_{300}$6%, $DP_{100}$4% materials.**

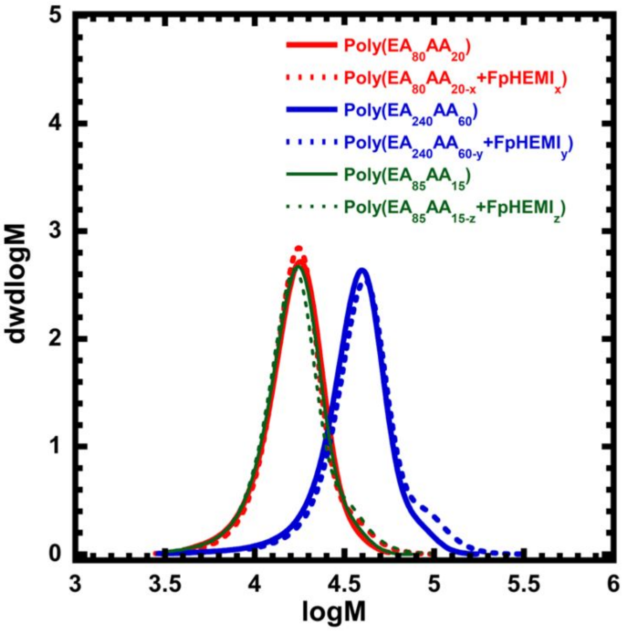


**Figure S4. Molecular weight distributions for all synthesized materials. Dispersity data is given in Table S1.**

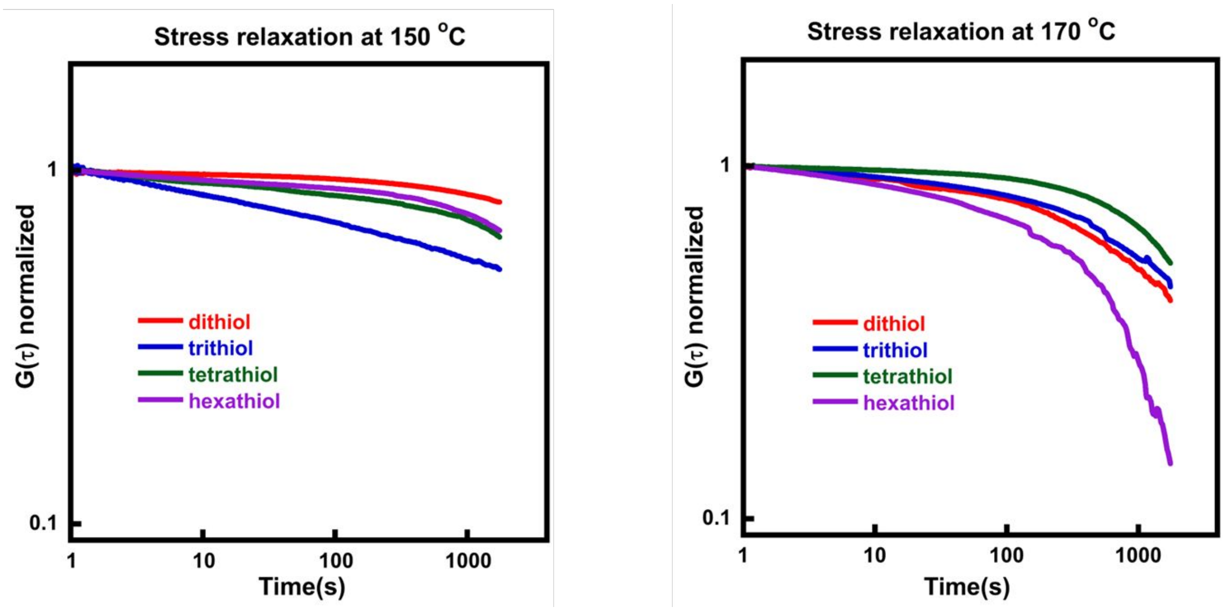


**Figure S5. Stress relaxation of the $DP_{100}$6% networks as a function of time. Data given in Table S5.**

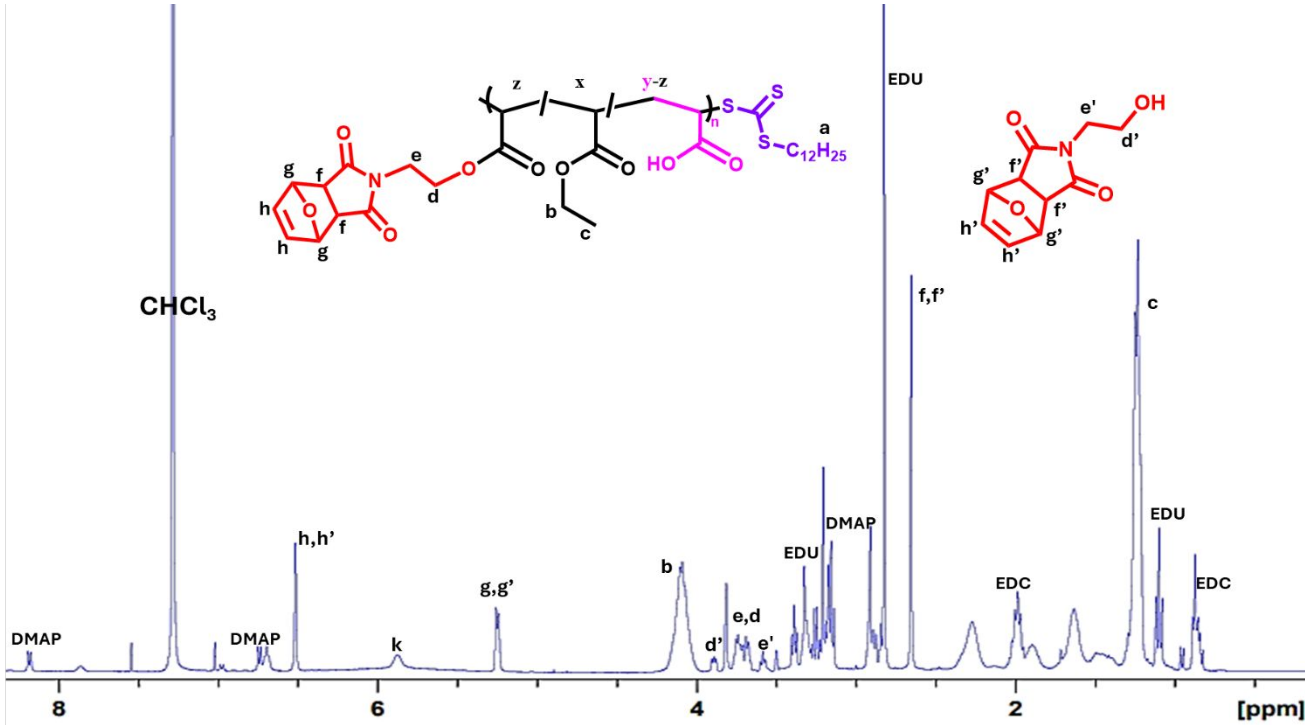


**Figure S6. $^1$H NMR spectra of the FpHEMI coupled polymer before precipitation for the purpose of analyzing the conversion yielded in the EDC coupling reaction(Table S1).**

## References:


(1) Zhang, B.; Digby, Z. A.; Flum, J. A.; Foster, E. M.; Sparks, J. L.; Konkolewicz, D. Self-Healing, Malleable and Creep Limiting Materials Using Both Supramolecular and Reversible Covalent Linkages. *Polym. Chem.* **2015**, *6* (42), 7368–7372. https://doi.org/10.1039/c5py01214g.

(2) Dodo, O. J.; Petit, L.; Dunn, D.; Myers, C. P.; Konkolewicz, D. Thermoresponsive, Recyclable, Conductive, and Healable Polymer Nanocomposites with Three Distinct Dynamic Bonds. *ACS Appl. Polym. Mater.* **2022**, *4* (10), 6850–6862. https://doi.org/10.1021/acsapm.2c00790.

(3) Fenimore, L. M.; Suazo, M. J.; Torkelson, J. M. Covalent Adaptable Networks Made by Reactive Processing of Highly Entangled Polymer: Synthesis-Structure-Thermomechanical Property-Reprocessing Relationship in Covalent Adaptable Networks. *Macromolecules* **2024**, *57* (6), 2756–2772. https://doi.org/10.1021/acs.macromol.3c02515.

(4) Lukichev, A. Physical Meaning of the Stretched Exponential Kohlrausch Function. *Physics Letters A* **2019**, *383* (24), 2983–2987. https://doi.org/10.1016/j.physleta.2019.06.029.
